# Online knowledge distillation network for single image dehazing

**DOI:** 10.1038/s41598-022-19132-5

**Published:** 2022-09-02

**Authors:** Yunwei Lan, Zhigao Cui, Yanzhao Su, Nian Wang, Aihua Li, Wei Zhang, Qinghui Li, Xiao Zhong

**Affiliations:** Xi’an Research Institute of High-Tech, Xi’an, 710025 China

**Keywords:** Computer science, Applied physics, Applied optics

## Abstract

Single image dehazing, as a key prerequisite of high-level computer vision tasks, catches more and more attentions. Traditional model-based methods recover haze-free images via atmospheric scattering model, which achieve favorable dehazing effect but endure artifacts, halos, and color distortion. By contrast, recent learning-based methods dehaze images by a model-free way, which achieve better color fidelity but tend to acquire under-dehazed results due to lacking of knowledge guiding. To combine these merits, we propose a novel online knowledge distillation network for single image dehazing named OKDNet. Specifically, the proposed OKDNet firstly preprocesses hazy images and acquires abundant shared features by a multiscale network constructed with attention guided residual dense blocks. After that, these features are sent to different branches to generate two preliminary dehazed images via supervision training: one branch acquires dehazed images via the atmospheric scattering model; another branch directly establishes the mapping relationship between hazy images and clear images, which dehazes images by a model-free way. To effectively fuse useful information from these two branches and acquire a better dehazed results, we propose an efficient feature aggregation block consisted of multiple parallel convolutions with different receptive. Moreover, we adopt a one-stage knowledge distillation strategy named online knowledge distillation to joint optimization of our OKDNet. The proposed OKDNet achieves superior performance compared with state-of-the-art methods on both synthetic and real-world images with fewer model parameters. Project website: https://github.com/lanyunwei/OKDNet.

## Introduction

Haze reduces image visibility with low contrast and color changes, which results in poor performance of high-level computer vision tasks such as object detection, automatic drive, and image understanding. Hence, image dehazing technology, as a key prerequisite, has become a crucial subject in computer vision. Generally, the formation mechanism of hazy images can be modeled as the following atmospheric scattering model:

1 $$ I\left( x \right) = J\left( x \right)t\left( x \right) + A\left( {1 - t\left( x \right)} \right) $$

where $I$ represents images obtained under hazy conditions; $J$ represents restored hazy-free images; $x$ represents the pixel location. Moreover, $A$ and $t$ represent the atmospheric light and transmission map.

Obviously, Eq. (1) is an ill-posed problem, which means we cannot acquire haze-free images $J$ from hazy inputs $I$ directly since both *A* and $t$ are undetermined. To this end, some prior-based methods estimate transmission map and global atmospheric light via a statistical rule on haze-free images, including dark channel prior (DCP)^1^, color-lines prior (CLP)^2^, color attenuation prior (CAP)^3^, and non-local dehazing (NLD)^4^. These prior-based methods can achieve excellent dehazing effect, but tend to cause halos and color distortion since unilateral assumption cannot hold in various scenes.

To solve these problems, some learning-based methods^5–9^ estimate atmospheric light and transmission map more accurately by data driving. Moreover, some methods^10–12^ estimate transmission map and dehaze images via a type-2 fuzzy approach and a Z-score-based weighting function. These methods dehaze effectively but still cause some color or illumination distortion since the atmospheric scattering model is just an ideal equation, which cannot completely represent the formation process of real haze. Hence, some end-to-end learning-based methods^13–25^ have been proposed, which do not estimate above intermediate parameters, and directly build the mapping between clear images and hazy inputs to achieve dehazing. However, due to the huge gap exists between hazy images and clear images, these end-to-end methods need strong feature extraction ability, and always solve this problem by increasing network depth^13,14^ and feature scales^15–18^. Recent researches have demonstrated that these multiscale methods have poor generalization ability especially when applied to real scenes due to over-fitting in synthetic field and lacking of extra knowledge for training guiding. Hence, some innovative works adopt prior dehazed images (i.e., the dehazed images of dark channel prior) or features in the reconstruction of clear images to guide network training, which achieve better dehazing effect in various scenes.

**Figure 1:**
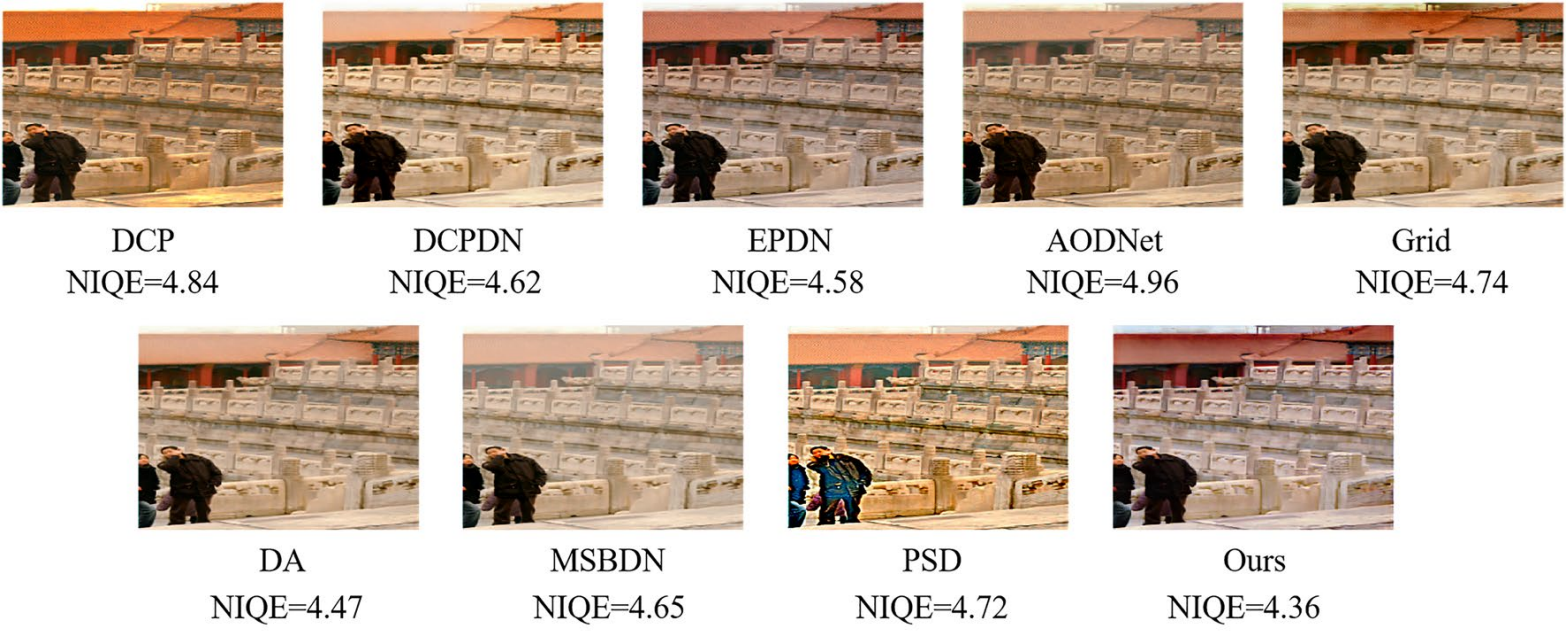
Comparative results on a real-world image using DCP, DCPDN, EPDN, AODNet, Grid, DA, MSBDN, PSD, and the proposed method. Natural Image Quality Evaluator (NIQE) is a non-reference criterion that is widely used in image quality assessment. Lower values of NIQE represent better performance.

**Figure 2:**
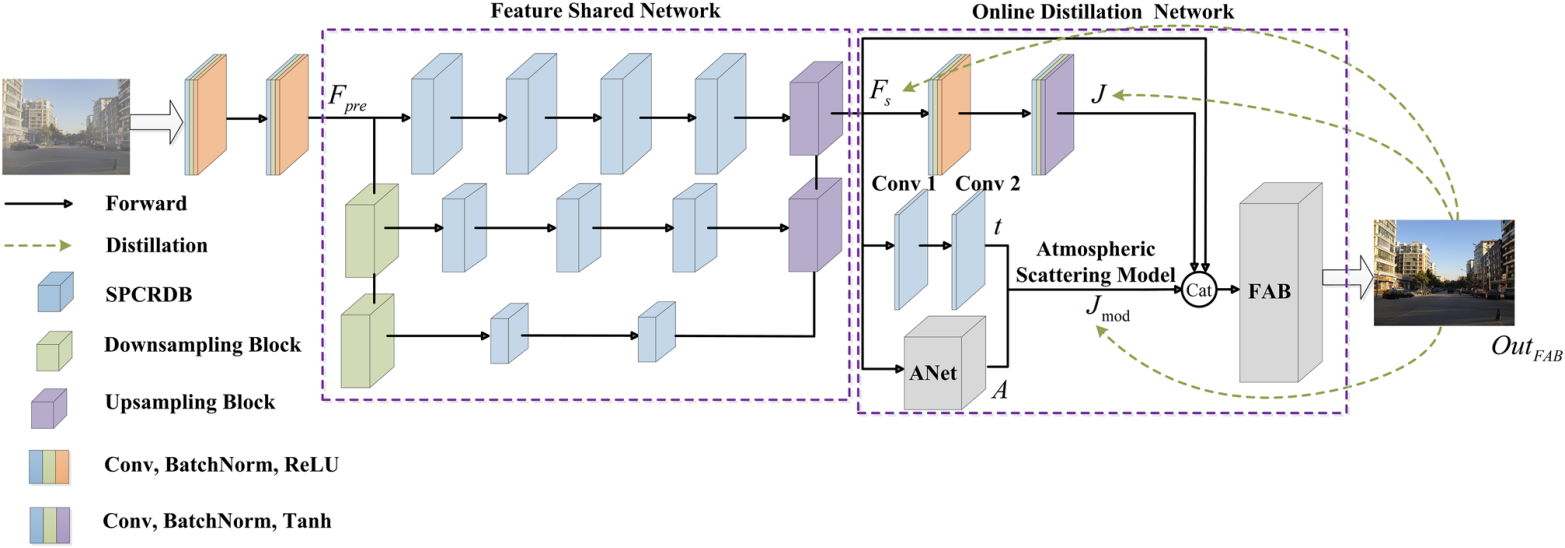
The general network structure of OKDNet.

Different from existing dehazing methods, we propose an online knowledge distillation network for single image dehazing named OKDNet. As shown in Fig. 1, we notice that model-based dehazing methods, including prior-based methods (i.e., DCP) and some learning-based methods (i.e., DCPDN), conduct better dehazing performance when compared to some recent multiscale end-to-end dehazing methods. For example, after DA, DCPDN achieves the best NIQE with a value of 4.62. Moreover, although DCP causes color distortion, it still achieves better values than AODNet in term of NIQE. This means the atmospheric scattering model, as a universal haze formation model, provides a training guidance and makes the network achieve better dehazing performance in real scenes although some distortion may be caused. Hence, the proposed OKDNet embeds the atmospheric scattering model in the network and adopts online knowledge distilling for optimization. As shown in Fig. 2, we firstly preprocess hazy images by two convolutions and acquire abundant features by feature shared network, a multiscale network constructed by some attention guided residual dense blocks (AGRDBs). Then these shared features are sent to online distillation network, where we achieve one-stage knowledge distillation by supervision training. Specifically, we get two preliminary dehazed images by the atmospheric scattering model and model-free method respectively. Moreover, considering that these two dehazed branches have complementary advantages in term of image contrast and color fidelity, an efficient feature aggregation block (FAB) is proposed to get a better dehazed image. In the online distillation network, the FAB performs as a teacher network, and two dehazed branches before the FAB work as students, which is optimized by building an extra loss function for knowledge distillation. Hence, different from traditional knowledge distillation, the proposed OKDNet do not need to pretrain teacher networks, which achieves the joint training and optimization of both teacher and student network. We call this knowledge distillation method as online knowledge distillation in this paper, and conclude main contributions as follows:

1. We propose an online knowledge distillation network for single image dehazing named OKDNet, which combines the merits of model-based dehazing methods and model-free dehazing methods by online knowledge distillation, and achieves favorable dehazing effect.
2. To improve the feature representation ability of the feature shared network, we introduce an attention guided residual dense block (AGRDB) to construct this multiscale network.
3. We propose a novel feature aggregation block (FAB) to effectively fuse the useful information of two dehazed branches and achieve a better dehazing effect.
4. We conduct experiments both on synthetic and real-world datasets to verify the dehazing effect of the proposed OKDNet, and prove the effectiveness of each module by an ablation study.

## Related work

### Model-based methods

Model-based methods restore dehazed images by atmospheric scattering model, where the estimation of atmospheric light and transmission map is the most critical issue. Early model-based methods adopt statistical assumptions concluded from haze-free images to estimate the transmission map and then recover haze-free images via atmospheric scattering model. For example, dark channel prior (DCP)^1^ assumes that clear images have low intensity in at least one channel, and acquires the atmospheric light and transmission map based on this theory. Color-lines prior (CLP)^2^ constructs a local formation model to recover the transmission map based on the lines offset, and accurately estimates transmission map. Moreover, Color attenuation prior (CAP)^3^ builds a linear relationship among color, haze concentration and scene depth, which achieves favorable dehazing effect. Another method NLD^4^ estimates the transmission map by hundreds of distinct colors, which greatly improves image visibility but tends to overenhance images. Early model-based methods have favorable dehazing effect but these dehazed images are contaminated by artifacts, halos and color distortion since unilateral assumption cannot hold in various scenes. With the development of deep learning, recent model-based methods tend to estimate transmission map and global atmospheric light by data driving. For example, MSCNN^5^ and DehazeNet^6^ build an efficient convolutional neural network (CNN) to estimate transmission map and restore visually pleasing results. However, the dehazing performance of these two methods is limited since the atmospheric light is still estimated by traditional methods. To solve this problem, AODNet^7^ combines the atmospheric light with transmission map into a parameter by setting a linear equation. DCNet^8^ constructs a dark channel network to estimate transmission map and dehazes images via the atmospheric scattering model. Another method DCPDN^9^ embeds the atmospheric scattering model into CNN, which directly restores dehazed images by the joint estimation of the atmospheric light and transmission map. These two methods alleviate cumulative error of two times parameter estimation. However, due to the atmospheric scattering model is a simplified model and cannot completely replace the formation process of haze, model-based methods still suffer from color and illumination changes. Differently, Sharma et al.^10^ use a type-2 fuzzy approach to estimate scene depth and global atmospheric light, and then dehaze images via the atmospheric scattering model. Moreover, they also propose a novel adaptive interval type-2 fuzzy filter^11^ as an AI agent, which can effectively retain the naturalness of results during the dehazing process. Another method^12^ estimates the scene transmission map and atmospheric light using Z-score-based weighting function for image dehazing.

### End-to-end methods

End-to-end methods, also called image translation, directly establish the mapping between hazy images and clear images instead of using atmospheric scattering model to achieve dehazing. Due to the huge discrepancy exists between hazy image domain and clear image domain, these methods often enhance feature extraction ability by increasing network depths and scales. For example, GCAN^15^ adopts smoothly dilation convolution to extract multiscale features, and merges these features by a gated network to alleviate artifacts. GFN^16^ derives three subimages (white balance (WB), contrast enhancing (CE), and gamma correction (GC)) from a hazy image, and directly recovers clear images by using learned confidence maps to fuse these three subimages. GridDehazeNet^17^ builds a deep multiscale network based on a grid architecture and enhances the information flow of different scales to recover haze-free images. Another method MSBDN^18^ constructs a multiscale network and combines the features from different scales using feature fusion mechanism to extract global and local features. Differently, EPDN^20^ acquires high contrast images based on the adversarial training between a pix2pixHD generator and a multiscale discriminator. However, due to trained on synthetic images and without any extra knowledge to assist training, above end-to-end methods conduct poor model generalization in real scenes. Thus, Shao et al.^21^ firstly propose the domain adaptation problem, and build a bidirectional translation network to dehaze effectively when applied to real-world hazy images. Chen et al.^22^ use physical priors to guide network training and proposed PSD network, which acquires high contrast results in multiple real scenes. Wang et al.^23^ propose a novel simple but powerful atmospheric illumination prior (AIP) to guide an end-to-end multiscale network training, which achieves good dehazing effect. Moreover, to reduce the rely of computational resource, Liu et al.^24^ propose a generic model-agnostic convolutional neural network to restore clear images from hazy inputs. CSIDNet^25^ establishes a compact single image dehazing network composed of three convolutional layers and improves its real-time applications.

### Knowledge distillation

Knowledge distillation is widely used in recent image super-resolution tasks, which aims to transfer the useful information of a cumbersome network to a designed light-weight network so that reducing parameters while maintaining the same performance. For example, Hong et al.^26^ applied knowledge distillation to heterogeneous task imitation, which adopts a complex dehazing network to guide the training of a simple dehazing network and achieves the same dehazing performance. Differently, Wu et al.^27^ proposed a training strategy for nonhomogeneous tasks, which adopts features generated in the process of clear image reconstruction to guide the training of dehazing network. Recently, more forms of knowledge distillation emerge, which greatly promotes the development of computer vision tasks. For example, Zhang et al.^28^ proposed a mutual learning strategy, which constitutes a mutual teaching and learning mechanism between two networks. Zhang et al.^29^ proposed a self-distillation strategy, which constructs a deep CNN and distills the features of deep convolutions to the shallower convolutions. Moreover, Li et al.^30^ proposed a novel online knowledge distillation method, which does not rely on pretrained teachers and improves the accuracy of pose estimation. Inspired by it, we build an online knowledge distillation network for single image dehazing named OKDNet. The OKDNet combines the merits of model-based methods and model-free methods, which acquires high quality dehazed images with discriminative textures and vivid color in both synthetic and real-world datasets.

## Proposed method

Existing end-to-end dehazing methods always acquire under-hazed results especially in real scenes due to lack of perception to haze. Considering that the atmospheric scattering model simulates the formation process of haze and conducts significant guiding function to network training, we propose an online knowledge distillation network for single image dehazing named OKDNet. As shown in Fig. 2, the whole architecture of OKDNet can be divided into three parts: preprocessing network, feature shared network, and online distillation network. The preprocessing network consists of two 3 × 3 convolutions to extract features of the input hazy images. These two convolutions followed by a batch normalization and ReLU function respectively, which change the channel numbers to 32, and maintain the image resolution to acquire preprocessed features $F_{pre}$.

### Feature shared network

To effectively extract haze-relevant features and acquire preliminary dehazed images, we send preprocessed features $F_{pre}$ to feature shared network. As shown in Fig. 2, the feature shared network firstly changes feature shape of $F_{pre}$ from 256 × 256 × 32 to 64 × 128 × 128 and further to 128 × 64 × 64 by two downsampling blocks to form features under different receptive field, Then, local features (e.g., structures and edges) and global features (e.g., color and textures) are extracted by this multiscale network. To improve the feature representation ability of each scale, we introduce an efficient attention guided residual dense block (AGRDB). Considering that low-resolution features contain more local textures information, we apply more AGRDBs to enhance feature extraction. After that, these enhanced features of adjacent scales are fused by channel concatenation, and restored to the former scale by two upsampling blocks until get the final shared features $F_{s}$ (256 × 256 × 32).

**Figure 3:**
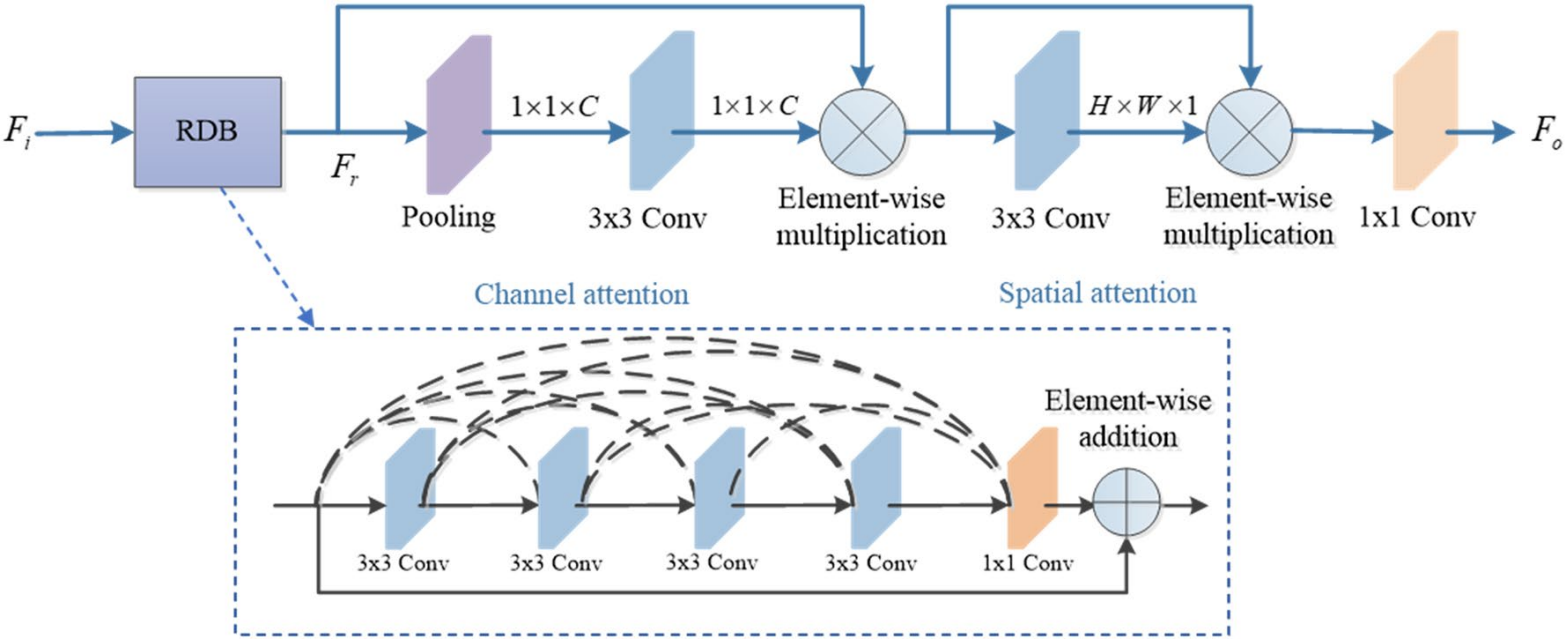
The structure of AGRDB.

As shown in Fig. 3, the proposed AGRDB firstly extracts features by a designed residual dense block (RDB). The RDB can effectively restore image details since the input of each convolution contains abundant features of all former convolutions. In our AGRDB, the RDB have the same configurations in^17^, which contains four 3 × 3 convolutions to extract features, and one 1 × 1 convolution to fuse these features before element-wise addition. After that, we adopt channel attention mechanism and spatial attention mechanism to guide the feature extraction of the AGRDB. In the channel attention mechanism, an average pooling is firstly used to compress feature maps $F_{r}$ after RDB to a channel vector (1 × 1 × C), and then a 3 × 3 convolution with a sigmoid function forms channel attention maps to weigh these input features by element-wise multiplication. After the channel attention mechanism, these refined features pay less attention to overenhanced features and effectively alleviate global color distortion. Considering that the haze distribution is uneven on image regions, a spatial attention mechanism is further applied to make the network pay more attention to haze-related pixels and high-frequency image regions. Different from the channel attention mechanism, the spatial attention mechanism directly acquires spatial attention maps (H × W × 1) by a 3 × 3 convolution combined with a sigmoid function, and weighs features by another element-wise multiplication. Finally, we further merge these feature maps and get the output $F_{o}$.

### Online distillation network

To effectively combine the complementary merits of model-based methods and model-free methods, we acquire two preliminary dehazed images based on these two methods and build an efficient feature aggregation block (FAB) for online knowledge distillation. As shown in Fig. 2, the online distillation network can be divided into two parts: student network and teacher network.

#### Student network

The student network is a multi-branch architecture consisting of two dehazing branches: model-free branch and model-based branch. In model-free branch, since the feature shared network has effectively extracted haze-relative features, we use two convolutions to generate dehazed image $J$, in which the first convolution is followed by batch normalization and ReLU function, and the second convolution is followed by batch normalization and Tanh function. For model-based branch, we use two convolutions to generate the transmission map $t$, and use the atmospheric light estimation network (ANet) in DCPDN^9^ to generate the atmospheric light $A$. By substituting the generated $A$ and $t$ into the atmospheric scattering model, another dehazed image $J_{\bmod }$ can be obtained.

$J$ and $J_{\bmod }$ are generated in different ways, and each has its own advantages: $J_{\bmod }$ has favorable dehazing effect but tends to cause color or illumination distortion, which may degrade the quality of dehazed images; by contrast, $J$ has better color fidelity but some local regions exist residual haze. Hence, we propose a FAB to merge the merits of model-based methods and model-free methods.

#### Teacher network

**Figure 4:**
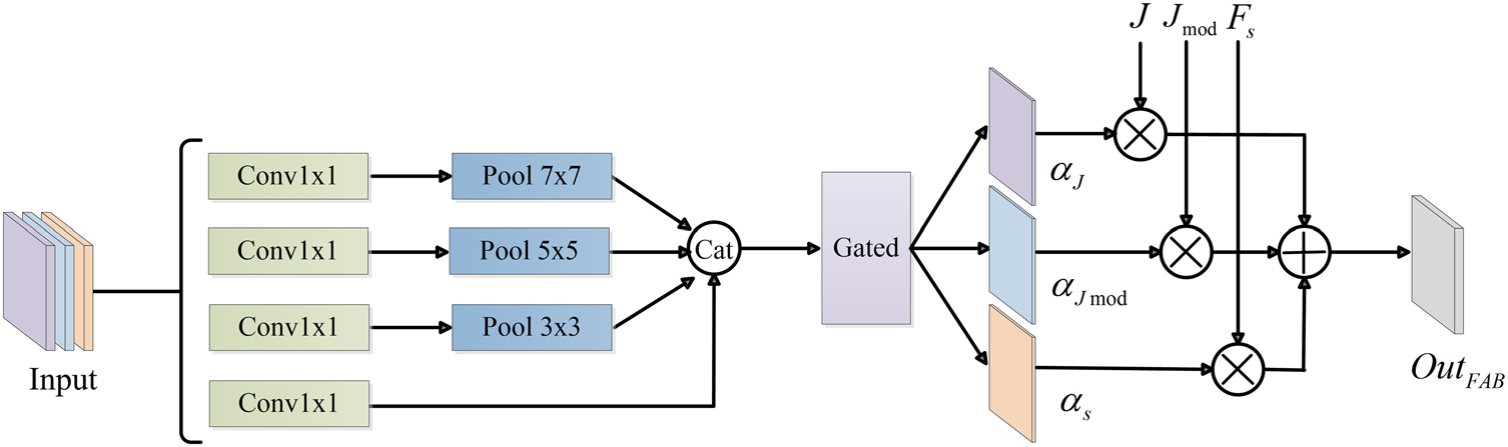
The structure of FAB.

Unlike traditional knowledge distillation methods, which use pretrained networks as teacher networks, we introduce FAB to combine the dehazed images of student branches to establish a powerful teacher network. As shown in Fig. 4, the FAB consists of four parallel point-wise convolutions (1$\times$1convolutions) and a gated network. Firstly, *J*, $J_{\bmod }$ and shared features $F_{s}$ are combined by channel-wise concatenation and used as the input of FAB, where $F_{s}$ contains rich features of original images. Then we adopt multiple parallel point-wise convolutions and pooling layers with different kernel sizes to extract local and global features simultaneously. In other words, a 1$\times$1 convolution with a 7 $\times$ 7 (5 $\times$ 5, 3 $\times$ 3) pooling layer is equivalent to directly using a 7 $\times$ 7 (5 $\times$ 5, 3 $\times$ 3) traditional parallel convolutions. Better than traditional parallel convolutions, the point-wise convolutions followed by pooling layers can reduce model parameters without using large convolutional kernel. Moreover, we concatenate multiscale features by channel-wise concatenation, and feed it into a gated network (a ${3}\times {3}$ convolution) to accurately weight features and generate three attention maps. These three attention maps ($\alpha_{J} ,\alpha_{J\bmod } ,\alpha_{s}$) weight input features ($J,J_{\bmod } ,F_{s}$) by linear combination to get an optimized dehazed image $out_{FAB}$, which can be expressed as Eq. (2):

2 $$ out_{FAB} = J*\alpha_{J} + J_{\bmod } *\alpha_{\bmod } + F_{s} *\alpha_{s} $$

where $\alpha_{J} ,\alpha_{\bmod } \, and \, \alpha_{s}$ are the weight maps generated by the gated network; $J$ and $\, J_{\bmod } \,$ are two dehazed images generated by student branches, $F_{s}$ is shared features before FAB.

As is mentioned above, the output of FAB combines the merits of dehazed images generated by model-free methods and model-based methods, which performs as a teacher to optimize these two dehazed branches (perform as students) in reverse. Moreover, considering that the shared features are essential for these two dehazed branches, we also set it as a student for optimizing. Hence, we adopt online knowledge distillation by building three extra distillation loss function between model-free dehazed images *J*, model-based dehazed images $J_{\bmod }$ and shared features $F_{s}$, which achieves the joint optimization of these three dehazed results (*J*, $J_{\bmod }$ and $Out_{FAB}$).

### Loss function

Several experiments^31,32^ have shown that the combination of pixel-wise loss and feature-wise loss can effectively improve network training. Hence, in our online knowledge distillation network (OKDNet), the overall loss function contains L1 loss, negative structural similarity (SSIM) loss^33^, and distillation loss, which can be expressed as Eq. (3):

3 $$ L_{loss} = L_{1} + L_{SSIM} + \lambda L_{diss} $$

where $L_{1}$ represents the L1 loss, $L_{SSIM}$ represents the negative SSIM loss, $L_{diss}$ represents the distillation loss, and $\lambda$ is a trade-off coefficient for balancing the overall loss, which is set to 0.5.

#### L1 loss

Previous research^13^ have shown that pixel-to-pixel losses can rapidly match the feature distribution between hazy images and clear images, thus we adopt L1 loss for network training. Different from L2 loss (mean square error), L1 loss (standard deviation error) can accelerate network training more stably, which can be expressed as Eq. (4):

4 $$ L_{1} = \left\| {GT - Out_{FAB} } \right\|_{1} $$

where $GT$ represents clear images and $Out_{FAB}$ represents dehazed images generated by feature aggregation block (FAB).

#### Negative SSIM loss

The negative SSIM loss can effectively match the luminance, contrast, and structure, and thus improves the structural similarity between clear images and dehazed images. Hence, we adopt negative SSIM loss to optimize the network training, which can be expressed as Eq. (5):

5 $$ L_{SSIM} = - SSIM(Out_{FAB} ,GT) $$

where $Out_{FAB}$ and $GT$ denote the dehazed images and clear images, respectively.

#### Distillation loss

In our OKDNet, the dehazed images obtained by FAB play the role of a teacher to teach feature shared network and students (two dehazing branches) in reverse. To achieve this, pixel-wise L1 losses are utilized to minimize the difference between dehazed images of teacher and student, which can be expressed as Eq. (6):

6 $$ L_{diss} = \left\| {Out_{FAB} - J} \right\|_{1} + \left\| {Out_{FAB} - J_{\bmod } } \right\|_{1} + \left\| {Out_{FAB} - F_{s} } \right\|_{1} $$

where $Out_{FAB}$ represents dehazed images generated by FAB (teacher), $J$ and $J_{\bmod }$ represent dehazed images of each branch (student), respectively, and $F_{s}$ represents the output of feature shared network. In this work, we first convert the size of $F_{s}$ to be the same as that of $Out_{FAB}$, and then calculate the loss between them.

## Experimental results

To evaluate the performance of our method on synthetic and real-world datasets, we quantitatively and qualitatively compare our OKDNet with several state-of-the-art methods, including DCP^1^, DCPDN^9^, EPDN^20^, AODNet^7^, Grid^17^, DA^21^, MSBDN^18^, and PSD^22^. All these methods are learning-based methods except DCP, which is a traditional prior-based method. The adopted datasets and implementation details are introduced in section "Datasets" and section "Implementation details", respectively.

### Datasets

During the training, we adopt the Indoor Training Set (ITS) in Realistic Single Image Dehazing (RESIDE)^34^ which contains 13,990 indoor hazy images and the corresponding haze-free images. For testing, we adopt three synthetic datasets (Synthetic Objective Testing Set (SOTS) in RESIDE, I-HAZE^35^, and O-HAZE^36^) to evaluate the performance of the proposed method. Among them, the SOTS contains 500 indoor and outdoor paired images produced by the atmospheric scattering model, and the I-HAZE and O-HAZE contain 35 paired indoor images and 45 paired outdoor images produced by professional haze machine. Moreover, considering that apparent discrepancy exists between synthetic and real-world hazy images, some real-world images in^2^ and URHI datasets^21^ are adopted to further verify the dehazing effect of these methods when applied in real scenes.

### Implementation details

The proposed method is trained and tested in the PyTorch framework. During the training, we resize the image to 256 × 256, set the batch size to 4, and train 30 epochs. Moreover, we use the Adam optimizer to accelerate the training process, and adopt a default value for the attenuation coefficient; where $\beta_{1} = 0.9$ and $\beta_{2} = 0.999$. We set the initial learning rate to 0.001, and decreases it to half after every five epochs.

### Comparisons with state-of-the-art methods

#### Results on synthetic datasets

**Figure 5:**
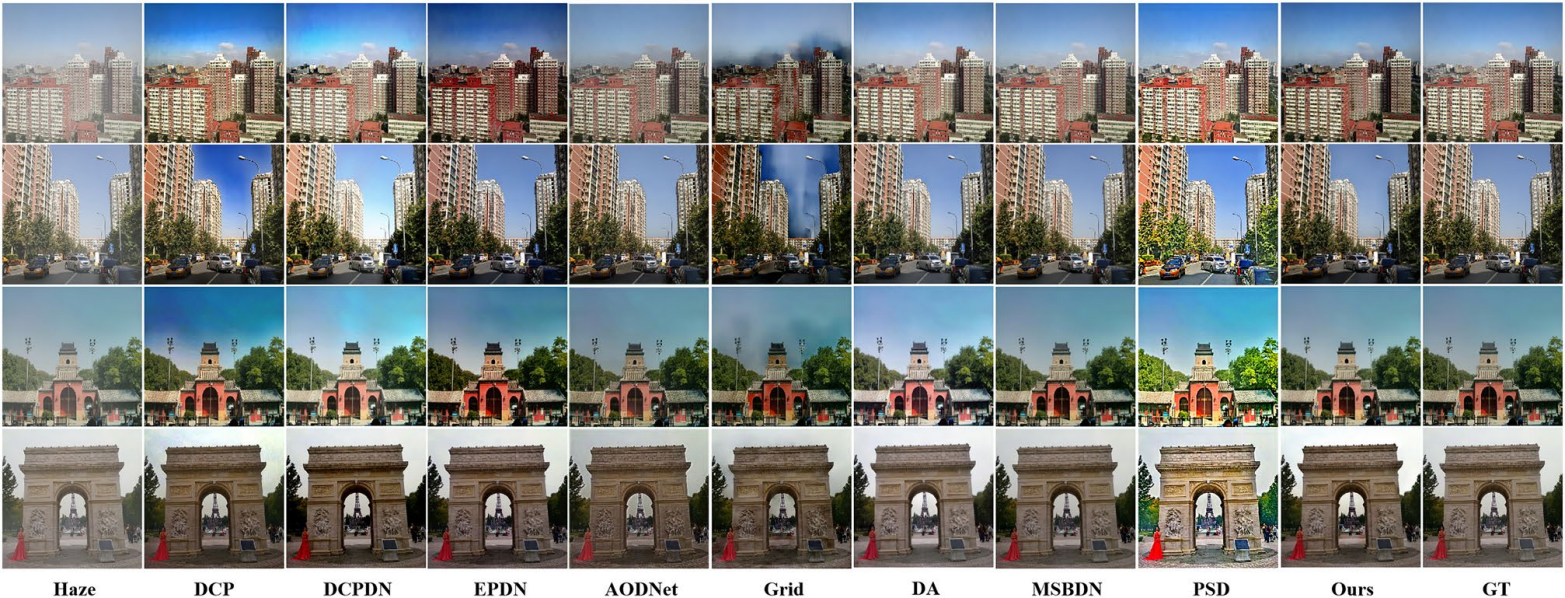
Results of comparison experiments on the SOTS outdoor datasets.

The atmospheric scattering model has shown the positive correlation of scene depth and haze concentration, which affects the haze removal effect of these algorithms. Hence, considering that these methods are trained on indoor images, we mainly test on outdoor images to verify the generalization ability. Figure 5 shows the experimental results of our method compared with some recent methods on the SOTS outdoor synthetic datasets. We can easily find that the prior-based method DCP suffers from color over-saturation and abnormal brightness since the unilateral assumption used to estimate the transmission map and atmospheric light is not applicable to various scenes. Moreover, the model-based DCPDN acquires high contrast results but causes some illumination distortion. By contrast, the Grid has overfitted in indoor scenes, which causes artifacts and leads to a large amount of residual haze in outdoor scenes. Moreover, the EPDN and AODNet dehaze images effectively but dim the brightness of dehazed images and degrade the visual effect of them. Differently, the DA causes slight brightness distortion during the dehazing process. And another method PSD improves the image contrast but leads to severe illumination over-saturation. Only MSBDN and our OKDNet acquire high quality results with discriminative textures and vivid color, which shows these two methods have better generalization ability in the changes of scene depth.

To further validate the performance of our OKDNet on synthetic datasets SOTS, five evaluation metrics (peak signal-to-noise ratio (PSNR), structural similarity (SSIM), lightness order error (LOE)^37^, naturalness image quality evaluator (NIQE)^38^, and model parameters) are employed for quantitative comparison. The results are shown in Table 1, where M is set as $1 \times 10^{6}$ for briefly representing the number of model parameters. For indoor datasets, DCP performs poorly with PSNR and SSIM being 19.95 dB and 0.872 respectively, which means that generated artifacts and color distortion severely influence the quality of restored images. Moreover, most learning-based methods acquire high PSNR and SSIM since they are trained on the indoor synthetic datasets. Only PSD performs poorly in indoor synthetic scenes due to the guidance of multiple priors. For outdoor datasets, we notice that the DCP achieves similar results to indoor scenes. By contrast, the PSNR and SSIM of these learning-based methods drop dramatically when applied to outdoor images due to the discrepancy of scene depth between indoor and outdoor scenes. Fortunately, with the help of embedding atmospheric scattering model, the proposed OKDNet improves PSNR from 23.16 dB to 23.38 dB and improves SSIM by 0.002 when compared with the second-best method MSBDN. Note that the MSBDN achieves good results on both indoor and outdoor images since it is trained by both indoor and outdoor images from RESIDE, while the proposed OKDNet is only trained by indoor images but achieves better results on both indoor and outdoor images, which proves its powerful generalization. For LOE and NIQE, the proposed OKDNet achieves the best value on both indoor images and outdoor images when compared with the other methods. Moreover, the proposed OKDNet also reduces the model parameters to 2.58 M and keeps excellent computational efficiency. According to the above evaluation metrics, the proposed OKDNet achieves the best results, which improves dehazing effect by merging the merits of model-based methods and model-free methods, and reduces model parameters by adopting online knowledge distillation.

**Table 1:** Quantitative comparison results on the SOTS outdoor datasets.

SOTS	Metrics	DCP	DCPDN	EPDN	AODNet	Grid	DA	MSBDN	PSD	Ours
Indoor	PSNR	19.95 dB	20.85 dB	25.09 dB	19.12 dB	*32.16 dB*	30.32 dB	**32.67 dB**	16.32 dB	30.92 dB
	SSIM	0.872	0.875	0.932	0.845	*0.984*	0.981	0.983	0.729	**0.988**
	LOE	227.94	331.51	258.30	266.54	188.55	247.55	*168.34*	320.59	**131.10**
	NIQE	4.385	4.801	4.618	4.106	4.231	4.830	4.839	*3.8310*	**3.319**
Outdoor	PSNR	20.44 dB	20.08 dB	20.31 dB	21.47 dB	16.21 dB	22.59 dB	*23.16 dB*	15.15 dB	**23.38 dB**
	SSIM	0.898	0.896	0.902	0.922	0.783	0.927	*0.936*	0.771	**0.938**
	LOE	205.45	148.121	338.77	277.79	337.83	116.72	*72.19*	314.94	**53.60**
	NIQE	3.1242	2.984	3.206	3.586	3.158	*2.897*	3.702	3.388	**2.849**
	Model Parameters	–	*0.94 M*	35.86 M	**0.09 M**	0.96 M	54.59 M	28.71 M	6.2 M	2.58 M

Number in bold, italics, and underline indicate the first, second, and third best results, respectively. DCP is a prior-based method without model parameters. Higher values of PSNR and SSIM; and lower values of LOE and NIQE represent better performance.

**Figure 6:**
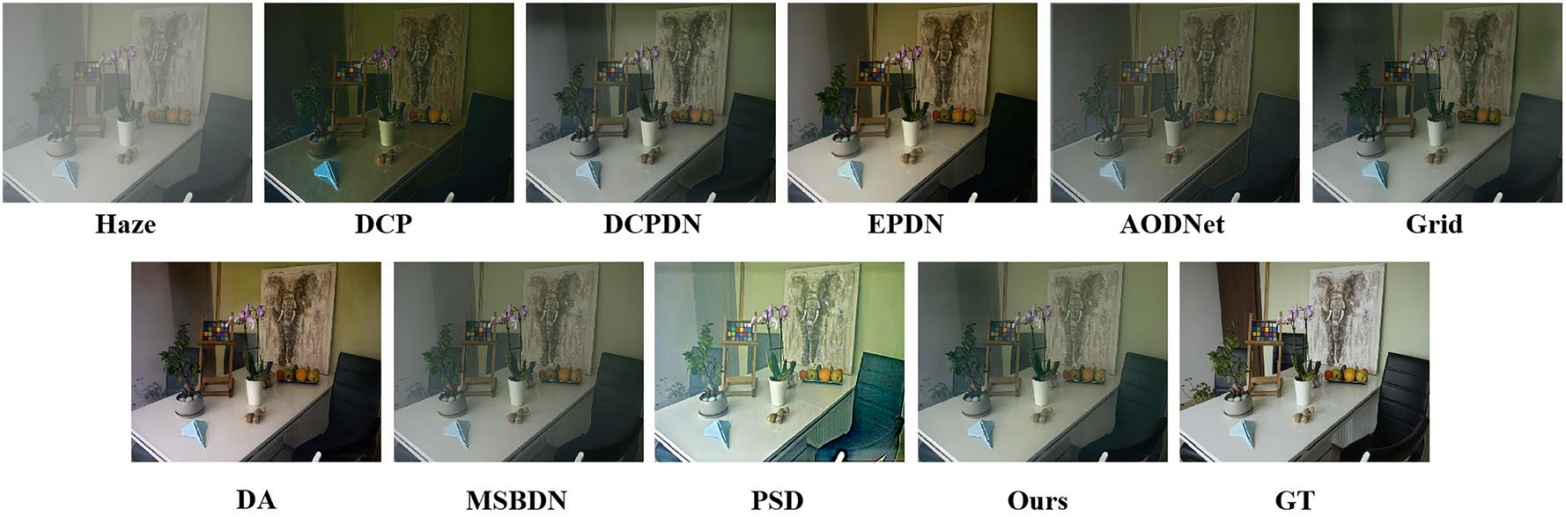
Results of comparison experiments on the I-HAZE.

**Figure 7:**
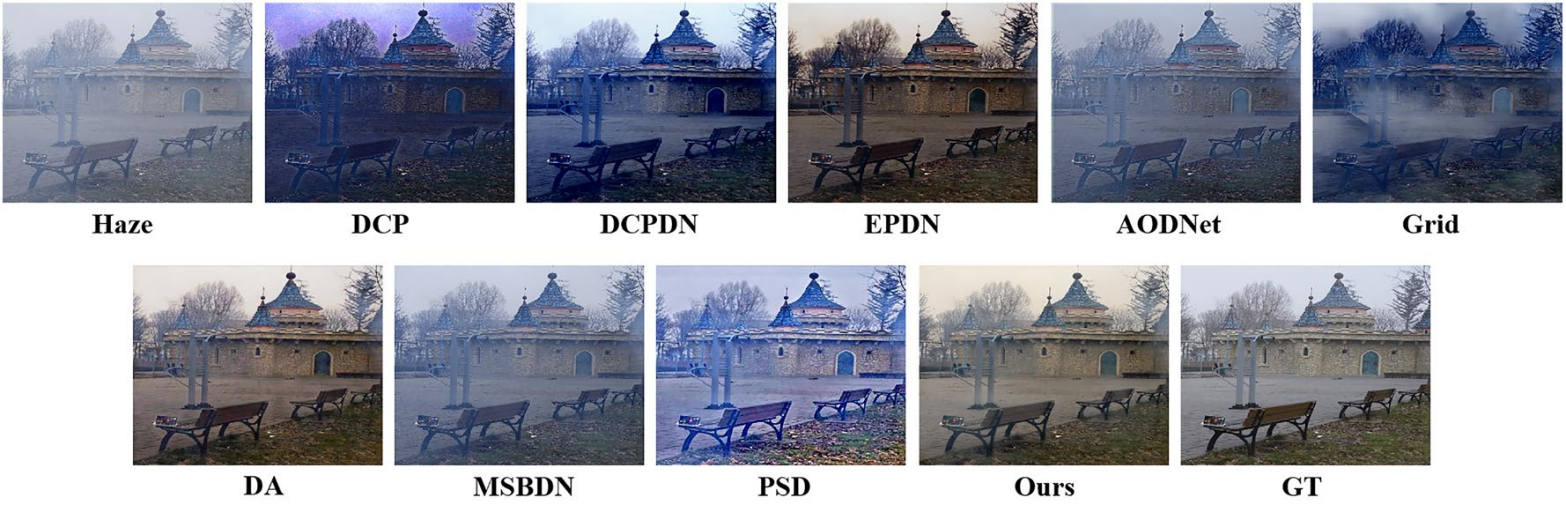
Results of comparison experiments on the O-HAZE.

To further validate the dehazing effect and generalization ability of the proposed method, we adopt I-HAZE and O-HAZE as benchmarks for qualitative and quantitative comparison. Figure 6 and Fig. 7 give the qualitative comparison on the I-HAZE and O-HAZE, and we can find that model-based methods DCP and DCPDN cause color distortion and darken the results. By contrast, model-free methods Grid and MSBDN tend to hardly deal with hazy images and leave residual haze in some regions. The PSD acquires dehazed images with high contrast but ovenhances the color, which makes the results look inauthentic. Only DA and the proposed method can effectively dehaze images and restore visually pleasing results. Moreover, better than the DA, the proposed OKDNet can restore more natural color and more clear textures. Table 2 gives the quantitative comparison results on the I-HAZE and O-HAZE. For I-HAZE, the DCP, DCPDN, and PSD perform poorly, which means that the abnormal illuminance and overenhanced color degrade the quality of dehazed images. Better than the other methods, the proposed OKDNet achieves the best values of PSNR, LOE, and NIQE being 17.16 dB, 275.75, and 3.743. Note that the DCPDN achieves the best value of SSIM being 0.826, which further verifies the atmospheric scattering model plays an important role in image dehazing. For O-HAZE, the proposed OKDNet significantly improves the PSNR from 18.37 dB to 18.96 dB and improves the SSIM from 0.765 to 0.837, which validates that it owns powerful generalization ability. Moreover, the OKDNet achieves good values of LOE and NIQE, which means that the results generated by OKDNet retain favorable image naturalness.

**Table 2:** Quantitative comparison results on the I-HAZE and O-HAZE.

	Metrics	DCP	DCPDN	EPDN	AODNet	Grid	DA	MSBDN	PSD	Ours
I-HAZE	PSNR	12.31 dB	14.27 dB	15.86 dB	15.06 dB	13.01 dB	*17.10 dB*	16.73 dB	12.92 dB	**17.16 dB**
	SSIM	0.676	**0.826**	0.751	0.772	0.634	0.807	0.798	0.712	*0.814*
	LOE	453.87	422.31	397.41	534.94	462.81	*303.67*	309.10	388.65	**275.75**
	NIQE	4.738	4.683	4.517	*3.993*	4.841	6.734	5.868	4.335	**3.743**
O-HAZE	PSNR	14.94 dB	13.79 dB	16.23 dB	16.61 dB	13.83 dB	*18.37 dB*	18.08 dB	14.46 dB	**18.96 dB**
	SSIM	0.672	0.726	0.716	0.696	0.707	0.712	*0.765*	0.677	**0.837**
	LOE	560.00	337.03	260.21	464.47	538.58	*203.86*	332.23	453.27	**180.33**
	NIQE	3.132	3.330	*2.777*	**2.670**	2.798	6.056	4.524	3.813	2.789

Number in bold, italic, and underline indicate the first, second, and third best results, respectively. Higher values of PSNR and SSIM; and lower values of LOE and NIQE represent better performance.

#### Results on real datasets

**Figure 8:**
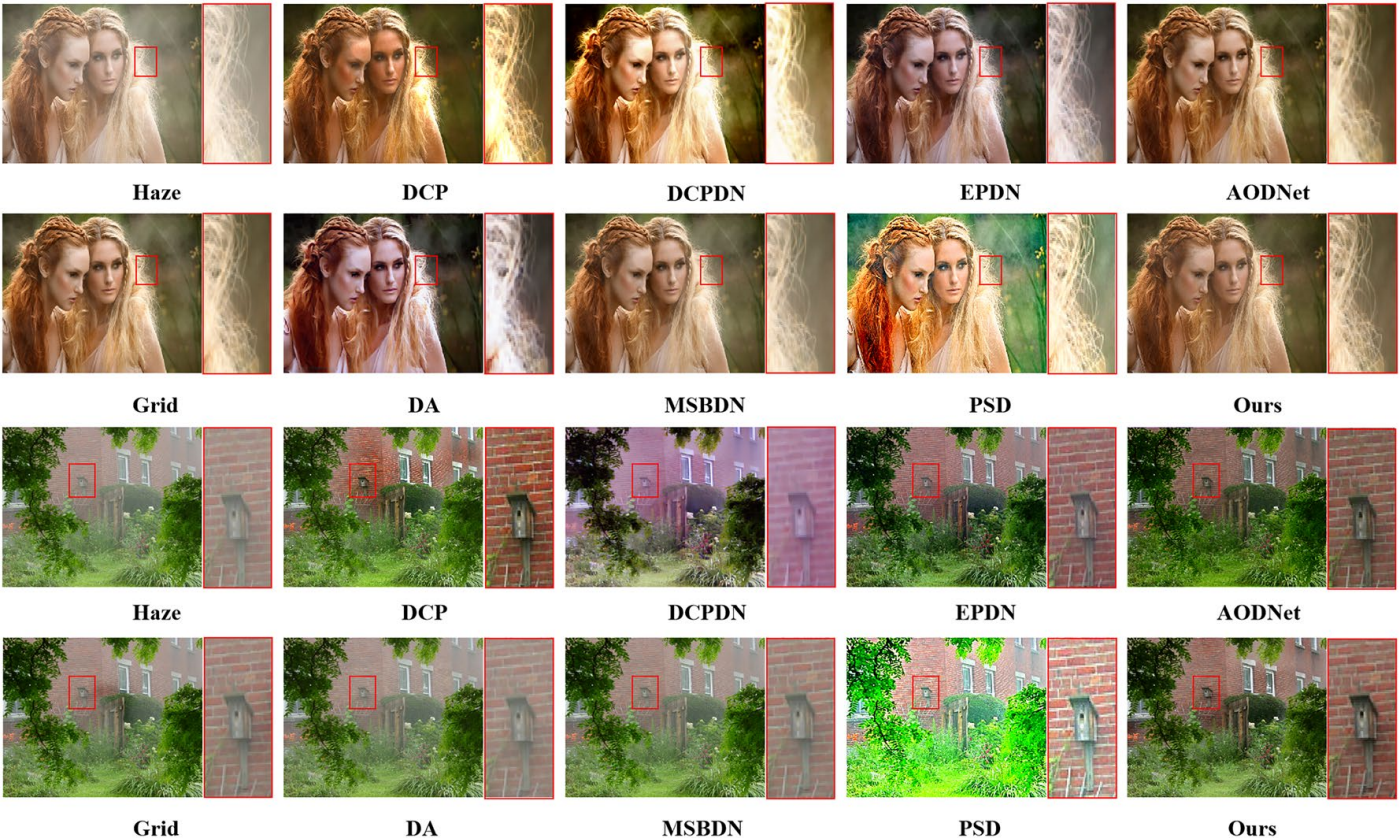
Experimental results of comparison on real images. The content in the red box in the image is enlarged for visual comparison.

Recent learning-based dehazing methods tend to present insufficient generalization ability and poor dehazing effect on real-world images since they are trained on synthetic datasets. Hence, several real-world hazy images from^2^ are selected to verify the performance of the proposed method when applied in real scenes. As shown in Fig. 8, prior-based method DCP tends to cause halos and severe color distortion, which validates that unilateral prior cannot hold in various scenes. Another model-based method DCPDN cannot effectively dehaze images and suffers from illumination distortion due to inaccurate estimation of the atmospheric light and transmission map. Moreover, the PSD acquires images with high contrast but tends to overenhance these images due to the guideline of multiple priors and atmospheric scattering model. By contrast, model-free methods acquire pleasing visually images but tend to dehaze ineffectively due to lacking of knowledge guiding. For example, the Grid and MSBDN methods perform poorly when applied in real scenes and lead to a large amount of residual haze, although they have good effect in synthetic datasets, which shows these two methods have over-fitted in synthetic scenes. Note that the MSBDN achieves bright results since it is trained by both indoor and outdoor images from RESIDE, while the proposed method is only trained by indoor images and consequently the results of the proposed method seem darker than MSBDN. Also, EPDN and DA acquire high quality images with better dehazing effect but tend to overenhance some regions. Compared with aforementioned methods, the proposed method and AODNet achieve better dehazing effect and recover more vivid color. Moreover, although the edges are not visible properly as in PSD, the results of the proposed method have advantages in restoring texture details (hair of woman) when compared with other methods.

**Figure 9:**
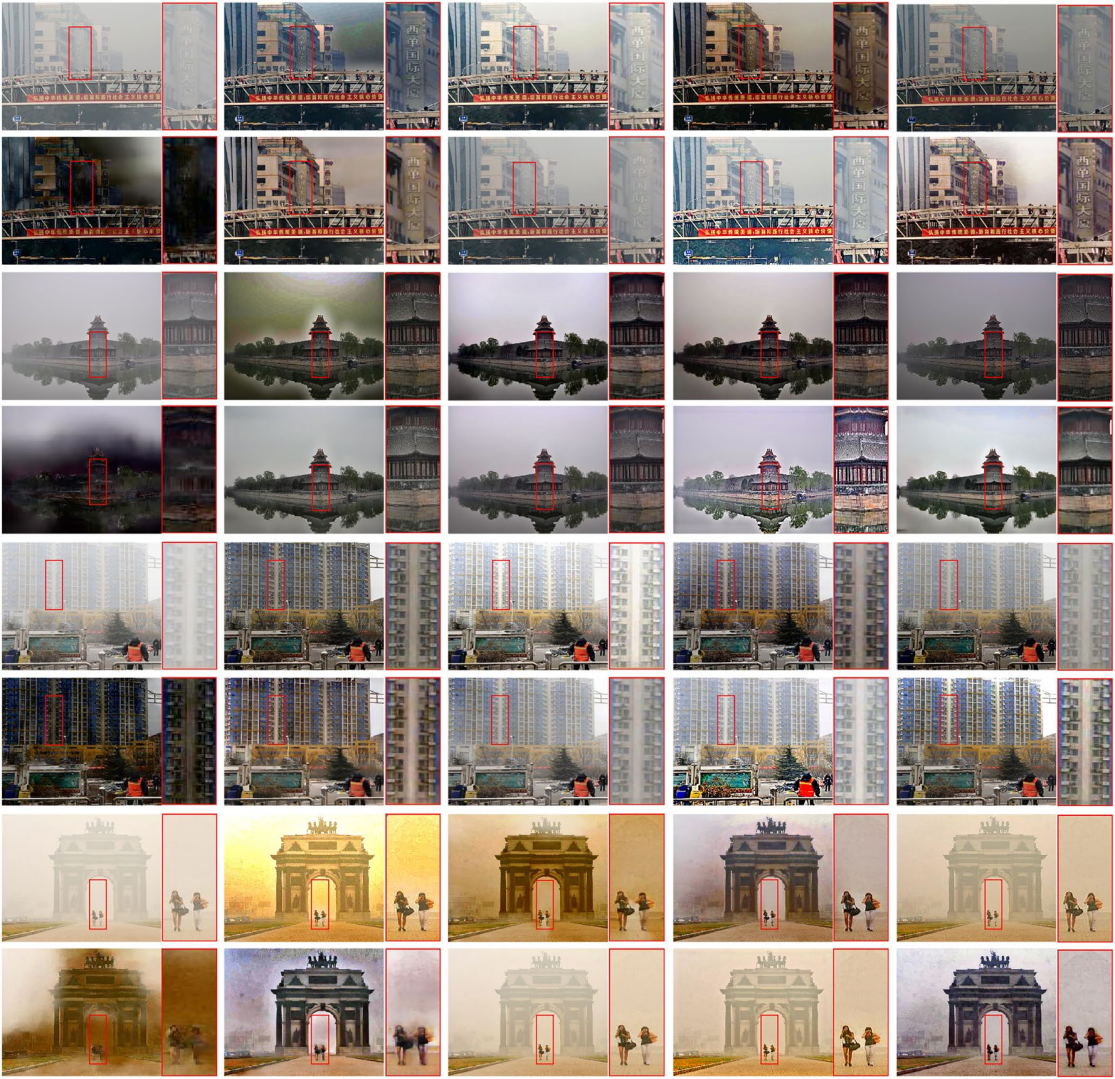
Results of comparison experiments on the URHI datasets. The content in the red box in the image is enlarged for visual comparison. From left to right and top to bottom: Input hazy images, dehazed images using DCP^1^, DCPDN^9^, EPDN^20^, AODNet^7^, Grid^17^, DA^21^, MSBDN^18^, PSD^22^, and the proposed method.

To further show the generalization ability of the proposed method, we compare these methods on real-world images in the URHI datasets^21^. As shown in Fig. 9, prior-based method DCP still effectively dehazes images, but leads to obvious color distortion. By contrast, learning-based methods cannot dehaze thoroughly. Specifically, model-based method DCPDN and AODNet cause illumination distortion and leave some residual haze in local regions. Moreover, Grid and MSBDN conduct poor dehazing performance in these scenes, which verifies these two methods have poor generalization in real scenes. Another method PSD also cannot dehaze effectively and tends to cause illumination and color changes. Only EPDN, DA and the proposed OKDNet achieve visually pleasing results and restore most textures. Unfortunately, the EPDN recovers high contrast images but exists severer color over-saturation. The DA fails to dehaze local haze and still causes slight color changes. By contrast, the proposed OKDNet acquires dehazed images with more distinctive features and more natural color by combining the merits of both model-based methods and model-free methods with online knowledge distillation.

For objective evaluation, we further make quantitative comparison on the real-world images from paper^2^ and URHI datasets by non-reference criterions that are widely used in image quality assessment since real-world images do not have corresponding ground truths. These criterions are Blind/Referenceless Image Spatial Quality Evaluator (BRISQUE)^39^, Natural Image Quality Evaluator (NIQE), and Perceptual Index (PI)^40^. All these criterions are aesthetic metrics, which can be used to evaluate the effect of haze, noisy, color shifts, illumination changes and other image degraded phenomenon. Thus, although above criterions are not designed to measure the effect of dehazing, they are widely used in dehazing fields since they can be used to compare the perceptual quality quantitatively. Table 3 gives the quantitative comparison results on the real-world images from paper1 and URHI datasets. For images in paper1, the proposed method achieves the best values of NIQE, BBRISQUE, and PI being 3.088, 13.05, and 2.015. Moreover, better than the second best AODNet, the comparison results further validate that the proposed method can more effectively restore dehazed image with natural color and good visual effect. For URHI datasets, the proposed method also achieves the best values when compared with other methods, which means that it has good generalization ability and is effective when applied in different scenes.

**Table 3:** Quantitative comparison results on the images in paper^2^ and URHI datasets.

Datasets	Metric	Haze	DCP	DCPDN	EPDN	AODNet	Grid	DA	MSBDN	PSD	Ours
Images in paeper^2^	NIQE	3.783	3.521	4.201	3.392	*3.299*	3.938	4.499	4.003	3.835	**3.088**
	BRISQUE	18.96	*13.74*	18.97	14.62	15.49	17.59	14.47	15.36	16.59	**13.05**
	PI	2.665	2.323	2.683	2.264	*2.242*	2.561	3.697	2.592	3.2048	**2.015**
URHI	NIQE	4.715	3.982	4.058	3.942	3.902	6.128	4.388	4.605	*3.822*	**3.539**
	BRISQUE	33.73	27.62	27.89	22.22	31.65	23.23	*21.79*	27.36	24.26	**19.36**
	PI	4.544	3.198	3.975	3.224	4.058	4.929	4.326	3.381	*3.194*	**3.038**

Number in bold, italics, and underline indicate the first, second, and third best results, respectively. Lower values of NIQE, BRISQUE, and PI represent better performance.

According to the above qualitative and quantitative experiments and analysis, the proposed method has superior dehazing effect and sufficient generalization ability. However, it still causes over-enhancement on some images, and the details of the image are lost. (the contrast of the tree in the second image of Fig. 8 and the edges of humans in the last image of Fig. 9) This is because the proposed method emphasizes the generalization ability of the model in the training process, which leads to the over-enhancement appearance on some images. Although the generalization ability of the proposed method is improved, the method will inevitably cause over-enhancement on some images since the atmospheric scattering model is embedded. This is a disadvantage of this method, and also a common problem of most dehazing methods. We think the generalization ability of the model is more important for the dehazing tasks, and a little distortion is tolerable.

### Ablation study

To validate the effectiveness of the proposed OKDNet, we conduct an ablation study to evaluate the performance of the following key modules: attention guided residual dense block (AGRDB), feature aggregation block (FAB), multiscale feature shared network, knowledge distillation of the model-based student branch, and knowledge distillation of the model-free student branch. The following variants are constructed in our experiments: Variant A, the proposed method without the AGRDB (we replace it to RDB designed in paper^17^); Variant B, the proposed method without the FAB (we replace it to feature aggregation unit designed in paper^30^); Variant C, the proposed method without the multiscale feature shared network (we replace it to a single scale network consisted of AGRDBs ); Variant D, the proposed method without the knowledge distillation of the model-based student branch; Variation E, the proposed method without the knowledge distillation of the model-free student branch; Variation F, the proposed method. We train these variants on the ITS datasets for 30 epochs and test the trained variants on the SOTS outdoor datasets, I-HAZE, and O-HAZE, and conduct quantitative comparisons to evaluate the performance of each variant. As shown in Table 4, the proposed method achieves superior performance with PSNR and SSIM on both three datasets. Moreover, by comparing the Variant F and the corresponding variant, we can find that each of the above modules contributes to the dehazing performance of the proposed OKDNet.

**Table 4:** Results of ablation study. Bold values indicate better performance.

	Metrics	Variant A	Variant B	Variant C	Variant D	Variation E	Variation F
SOTS Outdoor	PSNR	22.18 dB	23.06 dB	20.64 dB	22.67 dB	23.21 dB	**23.38 dB**
	SSIM	0.902	0.924	0.765	0.912	0.925	**0.938**
I-HAZE	PSNR	15.66 dB	16.85 dB	14.38 dB	17.06 dB	16.71 dB	**17.16 dB**
	SSIM	0.696	0.774	0.702	0.801	0.749	**0.814**
O-HAZE	PSNR	17.04 dB	18.23 dB	16.25 dB	17.73 dB	18.54 dB	**18.96 dB**
	SSIM	0.738	0.811	0.742	0.784	0.803	**0.837**

## Conclusion

In this paper, we propose an online knowledge distillation network for single image dehazing named OKDNet, which merges the merits of model-based methods and model-free methods. Specifically, we dehaze images respectively by model-based methods and model-free methods based on the shared features extracted by a multiscale architecture consisted of Attention Guided Residual Dense Blocks (AGRDBs), and adopt an efficient Feature Aggregation Block (FAB) to get a better dehazed image. Moreover, an online knowledge distillation strategy is adopted, which use the features of final dehazed image (perform as teacher) to teach each dehazed branch (perform as student) in reverse, and achieve the joint optimization of the whole network. Different from previous knowledge distillation methods, the proposed OKDNet do not rely on a pretrained teacher network, and show excellent dehazing effect on both synthetic and real scenes with more computational efficiency.

## Data Availability

The data that support the findings of this study are available from the corresponding author upon reasonable request.
